# Involvement of multiple stressors induced by non-thermal plasma-charged aerosols during inactivation of airborne bacteria

**DOI:** 10.1371/journal.pone.0171434

**Published:** 2017-02-06

**Authors:** Nachiket D. Vaze, Sin Park, Ari D. Brooks, Alexander Fridman, Suresh G. Joshi

**Affiliations:** 1 Center for Surgical Infection and Biofilm, Drexel University College of Medicine, Philadelphia, Pennsylvania, United States of America; 2 School of Biomedical Engineering, Science and Health Systems, Drexel University, Philadelphia, Pennsylvania, United States of America; 3 A.J. Drexel Plasma Institute, Drexel University, Philadelphia, Pennsylvania, United States of America; Universite Toulouse III Paul Sabatier, FRANCE

## Abstract

A lab-scale, tunable, single-filament, point-to-point nonthermal dieletric-barrier discharge (DBD) plasma device was built to study the mechanisms of inactivation of aerosolized bacterial pathogens. The system inactivates airborne antibiotic-resistant pathogens efficiently. Nebulization mediated pre-optimized (4 log and 7 log) bacterial loads were challenged to plasma-charged aerosols, and lethal and sublethal doses determined using colony assay, and cell viability assay; and the loss of membrane potential and cellular respiration were determined using cell membrane potential assay and XTT assay. Using the strategies of *Escherichia coli* wildtype, over-expression mutant, deletion mutants, and peroxide and heat stress scavenging, we analyzed activation of intracellular reactive oxygen species (ROS) and heat shock protein (hsp) chaperons. Superoxide dismutase deletion mutants (*ΔsodA*, *ΔsodB*, *ΔsodAΔsodB*) and catalase mutants *ΔkatG* and *ΔkatEΔkatG* did not show significant difference from wildtype strain, and *ΔkatE and ΔahpC* was found significantly more susceptible to cell death than wildtype. The *oxyR* regulon was found to mediate plasma-charged aerosol-induced oxidative stress in bacteria. Hsp deficient *E*. *coli* (*ΔhtpG*, *ΔgroEL*, *ΔclpX*, *ΔgrpE*) showed complete inactivation of cells at ambient temperature, and the treatment at cold temperature (4°C) significantly protected *hsp* deletion mutants and wildtype cells, and indicate a direct involvement of hsp in plasma-charged aerosol mediated *E*. *coli* cell death.

## Introduction

Biological aerosols (Bioaerosols) are environmental aerosols that contain microorganisms in them. These bioaerosols can be very effective in transmitting diseases caused by those pathogens. In most heating, ventilation, and air conditioning systems (HVAC), the predominant method of controlling microbes is through particulate filters. These filters, though effective, do not inactivate microorganisms. They merely trap the organisms, which subsequently survive for long periods of time over filters. These microbes can even proliferate, and cause infection at distant places after dissemination [1]. Hence there is a need of a technology which can effectively inactivate microbes in a very short exposure time. Currently, many technologies are being investigated for this purpose, which include carbon nanotubes [2], UV radiation [3], electrostatic fields [4] and more recently, the use of microwave and heat treatment [5] and afterglow plasma [6]. Plasma technology, especially non-thermal plasma technology has been increasing used as a tool for disinfection and sterilization [7–9]. Most of these studies are focused on using plasma to treat surfaces. Plasma is used in many cases to inactivate odor causing Volatile Organic Compounds (VOC) [10]; including non-thermal plasma for the treatment of bioaerosols [11].

Earlier research carried out at Drexel Plasma Institute has indicated the effectiveness of dielectric-barrier discharge (DBD) plasma system. After the exposure of a millisecond and 10 seconds to the plasma, 1.5 logs and 5.5 logs reduction were observed. Further investigation on this rapid inactivation effect suggested that ozone produced by plasma was not the major inactivating agent in this system but other reactive oxygen species produced by direct plasma exposure were largely responsible for the inactivation [12]. Another study demonstrated that atmospheric plasma induces a significant inactivation of both *Bacillus subtilis* and *Pseudomonas fluorescens* containing bioaerosol [13]. In addition, an inactivation of fungus in aerosol is demonstrated, wherein the investigation involving Scanning Electron Microscope image analyses revealed rupture of fungal spore membrane [14]. Although such studies have made progress towards the development of plasma systems and their application in tackling problem of bioaerosols, there is negligible literature which examines the effect of plasma on cellular responses of bacteria, the mechanism-based study which is widely used in antibiotic and biocide research [15–18]. DBD plasma discharge produces many reactive species which are bactericidal in nature, including ozone, and short-lived and stabilized species [19]. Reactive oxygen species (ROS) are proposed as a major contributor to inactivation effect produced by many types of plasma [20–24]. In most plasma systems, ozone is produced in large quantities, which often poses difficulty in investigating the effect of the species which are generated in small amounts. In this study, a new device of DBD treatment was built, and tested inside the laboratory-scale model of an HVAC system for inactivation studies of airborne bacteria. The present study aims to investigate the interaction of bioaerosols with a single filament, point to point DBD, the survivability of bacteria, and common pathway of bacterial cell stress response. Bacteria have multiple pathways of defending against endogenous and exogenous oxidative stress. We hypothesize that plasma-activated aerosols create oxidative stress in air-borne bacteria upon contact, which leads to rapid membrane depolarization, membrane permeation and inactivation. This study investigates two major players of ROS defense pathways, the catalase and the superoxide dismutase system [19,25], and demonstrates that although plasma used is nonthermal in nature, it activates heat-shock protein genes by plasma-charged aerosols at ambient temperature but not at low (4°C) temperature. Both the systems are reportedly involved in defense against UV radiation as well [26]. Through the series of experiments involving wildtype, specific deletion mutants, gene overexpression studies, and the scavenger-mediated rescue, we have tried to understand the bacterial cellular responses and cell survival.

## Materials and methods

### Plasma device

**Fig 1:**
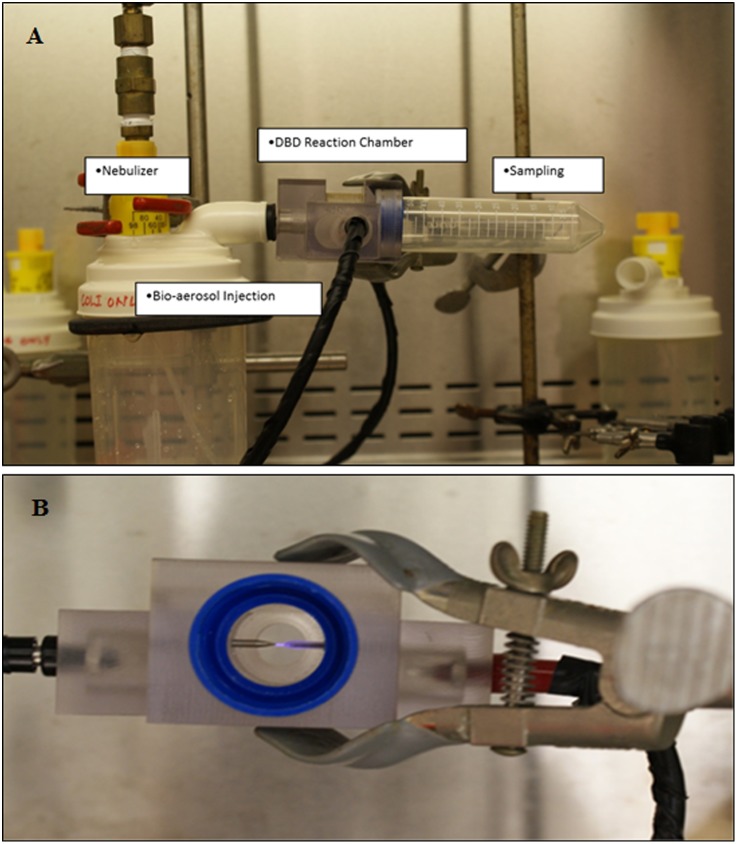
A customized miniature device of bioaerosol plasma treatment system. This system is tunable for rate of flow of bioaerosols, amount of bacterial load, plasma power and frequency, and thus for plasma energy dose. **(A)** The device picture showing parts of it. **(B)** A lateral view showing ignited plasma point to point discharge.

The setup used in present study consisting of an injection port, DBD plasma and bacteria treatment chamber, and a sampling system. The entire assembly is shown in Fig 1. A point to point discharge was used in DBD plasma. In brief, one electrode of the system was made of steel and the other was steel covered with quartz. The DBD was initiated between these two electrodes. The device was operated with an alternating current [10] pulsed power supply. The power supply consisted of a step up transformer that produces a high voltage between the output terminals. The microsecond plasma device produces a sharp discharge peak voltage, whose frequency can be adjusted. As per our earlier results related to air sterilization technology, the frequency was set at 1.5 kHz. This power supply has a range of 50–3500Hz frequency and 17.8–31.8kV p-p voltage, which translates to 1.4–18.2W Peak power. For the purpose of this study, the two settings chosen were 20kV Peak Voltage at 1.5 kHz which translates to 27.3 J/mL (Low Dose; at 2.1W peak) and 22kV peak Voltage at 1.5 kHz which translates to 41.6 J/mL (High Dose; at 3.2W peak). These voltage settings were chosen to be similar to our earlier experimentation [12]. The number of pulses per voltage period remains the same at all power levels as the frequency is the same. The energy density per micro-discharge would change as the peak voltage input to the electrodes is higher. Details of the paramemters on voltage, current, and pulse determined are shown in S1 Supporting Information. The inactivation efficacy was determined at tested energy levels for different loads of bioaerosol.

### Injection and sampling

For this study, a high volume Nebulizer (Teleflex Medical, Research Triangle Park, NC) was used. One hundred ml of the bacterial culture was used as the liquid for nebulization. The output of the nebulizer was modified to inject a fine stream of aerosol. The input to the nebulizer was connected through a filter to a cylinder of medical grade air (Airgas Inc., Radnor Township, PA). Tests were performed using optimized pressure, and 10 psi was chosen as the input pressure for all tests. After passing through the plasma zone (plasma-bioaerosol contact time, 0.13 millisecond), the bioaerosols were collected at the sampling port. The major methods for collecting bioaerosol particles were impingement and impaction [27–29]. Here, due to the geometry of the setup, impaction method was used. A 50 ml sterile conical tube (Fisher Scientific International, Inc., Hampton, NH) was connected to the port and the treated bioaerosols were allowed to be deposited on the inside wall of this tube. Ten ml of deionized water (EMD Millipore, Billerica, MA) was then added to the tube and mixed thoroughly to collect the bioaerosol particles. This suspension was used as the sample for colony counting. Two different nebulization times were used to achieve effect bacterial load. The optimized 30 seconds of nebulization (shorter time) and 180 seconds of nebulization (longer time) gave about 4 logs and 7 logs of colony forming units (CFU/ml). The 4 logs CFU were mostly used for proof-of-concept experiments and the 7 logs CFU were used for cell-density dependent inactivation confirmation and mechanisms of inactivation studies.

### Bacterial strains

Environmental aerosols have been shown to contain the large number of individual species of bacteria affecting health [30]. Therefore, the organisms relevant to a hospital setting were selected for the proof-of-concept study of the system. These includes *Acinetobacter baumannii* ATCC19606, *Escherichia coli* ATCC29522, *Staphylococcus aureus* ATCC29523, *Staphylococcus epidermidis* ATCC12228 (as representatives of Gram-positive and Gram-negative bacteria), and two antibiotic-resistant strains of methicillin-resistant *S*. *aureus* (MRSA) BBA1680, USA300 and BBA1683, USA400 [31,32]. *E*. *coli* is used as a model organism in various studies related to effect of biocides and plasma, and to characterize cellular pathways. The strains of *E*. *coli* wildtype and mutants used in mechanism-based studies are shown in S1 Table. These strains have been previously used to determine the role of ROS in the action of antibiotics [33], or involvement of heat-shock proteins. The regulon *oxyR* regulate the response to oxidative stress, and play a critical role in the activation of ROS defense mechanisms [34,35]. The strains (one each) of over-expressers of *oxyR* and deletion mutant of *oxyR* regulon were also used in these studies [36]. The strains of *hsp* gene deletion mutants were purchased from *E*. *coli* Genetic Stock Center, Yale University, New Haven, CT, USA.

### Colony count assay

As mentioned above two different DBD plasma energy doses and two different nebulization times (two different net concentrations of CFUs) were tested to investigate whether DBD plasma inactivates aerosolized bacteria (bioaerosol). Initially, *E*. *coli* wildtype was tested for colony count assay to demonstrate the level of inactivation. The collected samples were diluted with deionized water and 100μl of each sample was spread over Tryptic Soy Agar (TSA) plate and incubated at 37°C for 18 to 24 hours. The plates were observed the next day and the colonies counted. The number of surviving bacteria in original sample was calculated by multiplying the number of colonies to the number of dilutions performed. Datasets were analyzed using Microsoft Excel and verified using GraphPad Prism 4 software. Both the untreated bioaerosols and DBD treated bioaersols were compared for inactivation efficacy. Similarly other bacteria and mutants were tested to determine log reduction of CFUs.

### Measurement of cell membrane potential

The BacLight Bacterial Membrane Potential Kit (Cat # B34950, Molecular Probes, Inc., Life Technologies Corporation, Grand Island, NY) was used for flow cytometric measurement of membrane potential of untreated versus plasma treated bioaerosls. A Guava Flow Cytometer and Guava ExpressPlus assay software (EMD Millipore, Billerica, MA) were employed for the assay, following the direction of manufacturer. The assay employ solution of carbocyanine dye DiOC_2_(3) (3,3’-diehtyloxa-carbocyanine iodide). DiOC_2_(3) exhibits green fluorescence in all bacteria, but the fluorescence shifts toward red emission as the dye molecules self-associate at the higher cytosolic concentrations caused by larger membrane potential. Therefore a fluorescence ratio (red/green) is calculated to demonstrate any depolarization of membrane by comparing experimental conditions versus control (untreated) cell samples. The following formula was used to determine the number of bacteria in the sample:

$$\text{Bacteria}/\text{mL}=\left(\left(number\;of\;events\;in\;bacteria\;region\right)\times \;\left(dilution\;factor\right)\right)/\left(number\;of\;events\;in\;bead\;region\right)\times 10^{-6}\;)$$

Finally, the fluorescence ratios (red/green) for various treatment conditions were graphed.

### LIVE/DEAD BacLight bacterial cell viability assay

The bioaerosols either untreated or treated with DBD were prepared as described above (under ‘Injection and Sampling’), and cell pellet was obtained by centrifugation at 5000 revolutions per minute for 5 minutes and after washing twice with sterile PBS solution the samples were processed for LIVE/DEAD assay following the direction of manufacturer and as previously described [9]. The images were captured from 5 random fields for three different sets of experiments. The saved TIFF images were edited using Adobe Photoshop CS3 and analyzed using ‘ImageJ’ program (NIH, Bethesda, MD) to calculate mean number of green and red pixel in each area; and graphed using Microsoft Excel Program.

### XTT assay for cellular respiration and cell proliferation

The untreated or plasma treated bioaerosol samples processed as above were subjected to 2,3-bis-(2-methoxy-4-nitro-5-sulfophenyl)-2H-tetrazolium-5-carboxanilide (XTT) assay, following the described method [8,23,37]. The readings were normalized, and percent surviving (respiring) cells were calculated against untreated samples. In parallel, colony count assay was performed for comparison.

### Influence of antioxidants on survival of cells exposed to DBD as bioaerosol

In addition to deletion or overexpression mutants *E*. *coli*, the cells also challenged pre- and post- DBD-exposure of bioaersols to specific scavengers of reactive oxygen species. D-mannitol is a sugar that has been shown to defend peroxidative stress and hydroxyl radical-induced stress [23,38,39], while thiourea is an organosulphur compound that has been used as ^.^OH scavenger [40,41]. Catalase (Sigma-Aldrich, St. Louis, MO) was obtained in lyophilized powder form and reconstituted into Phosphate Buffer (50 mM Potassium Phosphate Buffer, pH 7.0 at 25°C). Both, mannitol and thiourea (Sigma—Aldrich) were acquired as a powder and reconstituted in deionized water, and the solutions were filter sterilized (0.22 uM filter), prepared aliquot and either used (10 mM final concentration) as fresh or stored at -20°C for further experiments. Various concentrations of catalase (50 to 500 units) were tested during pre- and post- DBD exposure challenge experiments.

### Influence of cold temperature on cell survival

*E*. *coli* wildtype and deletion mutants of specific chaperon/ heat-shock protein were investigated to study the effect of DBD on bioaerosol at room temperature and at 4°C. In brief, the bacterial strains were aerosolized and allowed to pass through DBD plasma as mentioned above. The whole treatment and collection of post-treatment samples were harvested either at room temperature or at 4°C. The bioaerosol samples, either untreated (control) or treated with DBD were serially diluted and colony count assay performed as described above.

### Statistical analyses

The differences among strain susceptibilities to plasma-charged aerosols or hydrogen peroxide (internal control) were calculated using Student *t-*test by direct comparison with untreated controls (control bioaerosol) or with the corresponding condition of with or without scavenger on log of Colony Forming Units. Experiments with wildtype and mutants were run in parallel, and the experiments used as a pair-wise measure for a one-tailed comparison with *n* representing the number of replicate experiments. *P* value of < 0.05 is considered as a significant change.

## Results

A miniaturized, point-to-point single filament DBD plasma device designed for this study is shown in Fig 1. Different parts of this device such as bioaerosol infection unit, nebulizer, DBD reaction chamber, and sampling collection units are also visible in Fig 1.

### Inactivation of bacteria in bioaerosols

**Fig 2:**
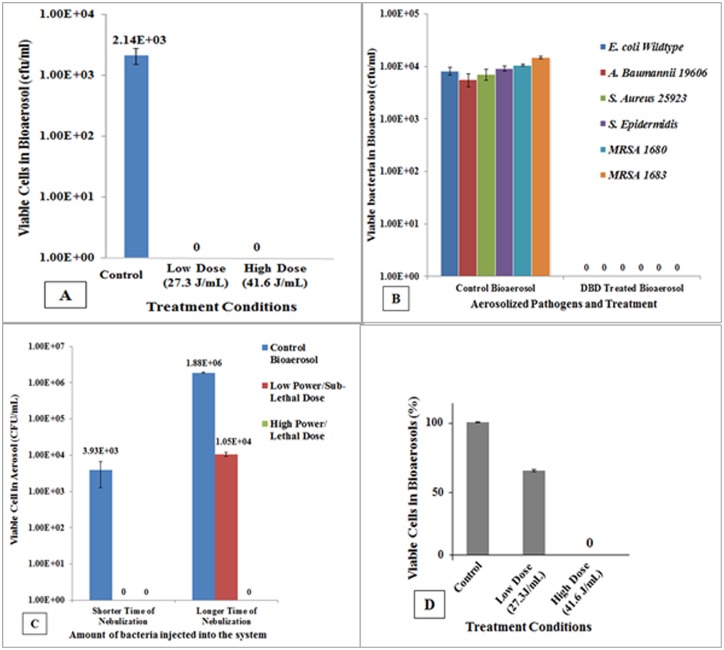
Plasma energy and airborne bacteria inactivation responses to plasma charged aerosols. **(A)** Low bacteria load (~ 4 logs CFUs of *E*. *coli*) and their inactivation response to different plasma energy doses. **(B)** Responses of representative Gram-positive and Gram-negative, and antibiotics resistant bacteria in their aerosolized forms to Low plasma energy treatment, indicating a complete inactivation of low bacterial load (~ 4 logs CFUs). **(C)** Comparison of high bacteria load (~ 7 logs CFU) with low bacteria load (*E*. *coli*) against High Dose and Low Dose of plasma energy (which subsequently designated as lethal and sublethal plasma doses, respectively). **(D)** Viable cell count of *E*. *coli* cells as determined by BacLight Bacterial Viability Cell Assay confirming that Low Dose at 7 logs of CFUs acts as sublethal dose and does not completely inactivate cells whereas High Dose is lethal to cells.

To investigate whether bacteria in aerosolized form can be inactivated by the newly designed single filament, point to point DBD plasma device, we started with (pre-optimized nebulization time for) low number of bacteria (~1 x 10^4^ CFU/ml), and exposed to variable amount of plasma energy (J/mL), as described under material and methods. Fig 2A demonstrates that *E*. *coli* wildtype completely inactivated by Low Dose and High Dose DBD plasma-generated energy-exposure. Subsequently, we challenged other Gram-positive and Gram-negative representative pathogens, including the antibiotic resistant and biofilm producing methicillin-resistant *S*. *aureus* (MRSA). Fig 2B demonstrates that broad-spectrum of pathogens is inactivated when present in bioaerosol by Low Dose of DBD plasma energy exposure. In order to investigate whether higher bacterial load present in aerosols can also be effectively inactivated, we pre-optimized nebulization time which releases ~ 7 logs CFU, and challenged to Low Dose and High Dose of plasma energy. Fig 2C is a graphical presentation of the responses of low and high bacterial loads to Low Dose and High Dose of plasma energy; and indicates that High Dose exposure completely inactivate bacteria (*E*. *coli*) when present in low as well as high bacterial loads, whereas Low Dose does not inactivate high bacterial load (~ 7 logs) completely. Low Dose plasma energy could give rise ~ 2 logs reduction, and hence, we referred it as sublethal dose of DBD plasma for high bacterial loads. Fig 2D represents the findings of LIVE/DEAD BacLight Cell Viability Assay, and confirms that Low Dose of DBD plasma energy is unable to inactivate 7 logs CFU of *E*. *coli*, and acts as ‘sublethal dose’ of DBD plasma for high load of cell concentration. This dependence on the bacterial load and plasma energy has also been observed in other types of plasma studies [33,42,43].

### Changes associated with sublethal dose (Low Dose) of plasma

The bioaerosols sample containing ~7 logs of CFU were either treated with Low Dose or High Dose of plasma energy or left untreated, and the entire sample was added to Tryptic Soy Broth (TSB) and incubated overnight at 37°C in stationary incubator and visually observed after 24h and 48h to make a comparison. S1 Fig depicting TSB tubes demonstrate no visible growth in the bioaerosol samples treated with High Dose of plasma energy, whereas Low Dose treated samples indicates turbidity (growth) in TSB (growth was confirmed subsequently on TSA plates; pictures not shown). These observations suggest that a sustained incubation of treated samples showed no recovery from lethal dose experiment and the Low Dose (27.3 J/mL) DBD treatment is appropriate to consider ‘sublethal’ dose. In parallel, we performed two additional tests which are indicators of membrane associated changes, the measurement of cell membrane potential, and the XTT assay which measure cellular respiration. S2A Fig demonstrates the significant changes in cell membrane potential as compare to untreated/ control bioaerosols, and indicates member depolarization. S2B Fig demonstrate the findings of XTT assay performed post-exposure of bioaerosols to sublethal (Low Dose) and lethal (High Dose) DBD plasma energy, and indicates that *E*. *coli* cells exposed to High Dose do not undergo dormancy, but actually completely inactivated whereas sublethal dose treated cells regrow, comparable to untreated (control) bioaerosol.

### Involvement of superoxide dismutase and catalase in DBD stress-induced cell death

**Fig 3:**
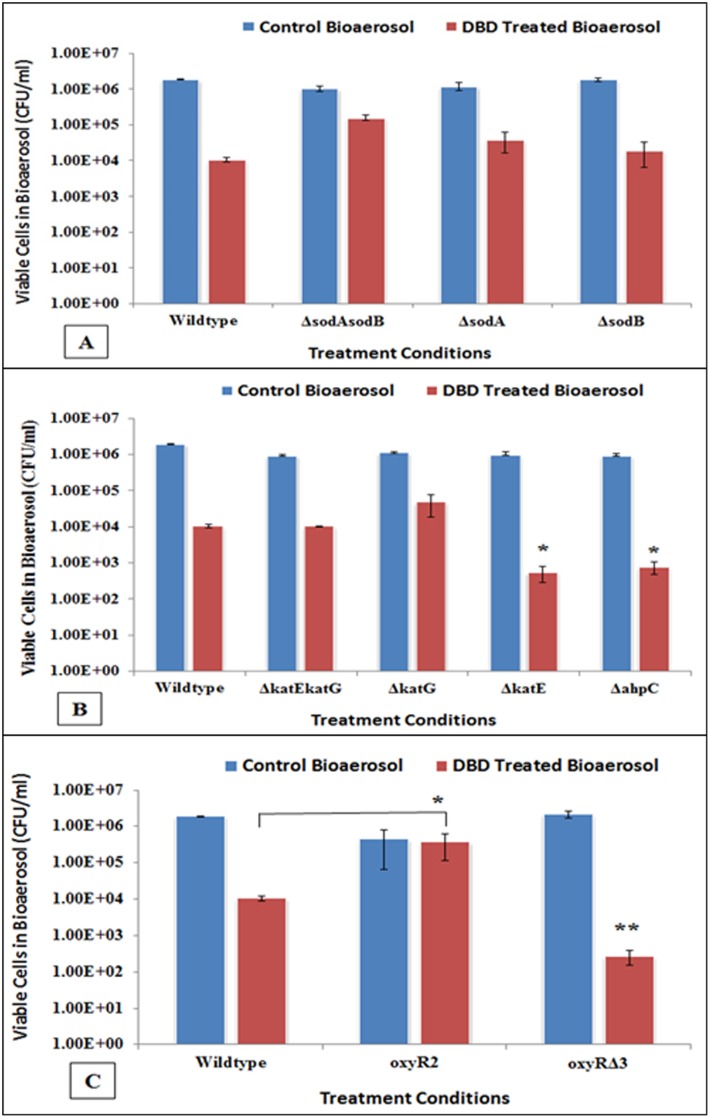
Colony assays demonstrating the relative susceptibilities of *E*. *coli* wildtype and its various derivatives to plasma charged aerosols. **(A)** Superoxide dismutase single deletion (*ΔsodA*, *ΔsodB*) and double deletion (*ΔsodAΔsodB*) mutants do not show significant difference in susceptibilities from wildtype cells. **(B)** Catalase/ hydroperoxidase deletion mutants demonstrates that *katE* and *ahpC* are significantly more susceptible to plasma charged aerosols as compared to wildtype (*), whereas *katG* and double mutant (*katEkatG*) behaved like wildtype and do not show significantly different susceptibility from wildtype. **(C)** The responses of *oxyR* regulon demonstrate that its deletion mutant is significantly more susceptible (**), and overexpression (*) mutant is more resistant to plasma charged mediated inactivation as compared to wildtype, indicating direct involvement of *oxyR* in inactivation mechanism.

To investigate the susceptibility profile of *E*. *coli* deletion mutants of superoxide dismutase (sod) toward cellular stress generated by DBD plasma-charged aerosols as compared to wildtype strain, the strains in bioaerosolized form were exposed to DBD. The results of the *sod gene* knockouts are shown in Fig 3A. The inactivation pattern of the *sod* single gene knockouts was not significantly different than the wildtype (p = 0.0943 for *ΔsodA*, p = 0.3088 for *ΔsodB*), and remained within or less than 1 log reduction. The double knockout strain *ΔsodAsodB* actually showed greater survival as compared to the wildtype (p = 0.0004). Similarly, the single and double knockouts of *kat* were tested and the results are shown in Fig 3B. For double knockout *ΔkatEΔkatG*, there was no significant difference in susceptibility to inactivation when compared to wildtype (p = 0.7017). The *ΔkatG* also showed no significant difference but *ΔkatE* however, was significantly more susceptible to inactivation as compared to wildtype (p = 0.0002). The inactivation observed was 1.5 logs greater than in wildtype. This was the first indication of the involvement of peroxidative stress. The alkyl hydroperoxidase is another system involved in defense against peroxidative stress, and hence *ΔahpC* was tested to plasma exposure. The inactivation of this strain was similar to the *ΔkatE* and significantly greater than wildtype (p = 0.0003). Thus, two strains which are deficient in defending against peroxidative stress were shown to be susceptible to plasma discharge. The *ahpC* gene is part of the *oxyR* regulated system. The system was then tested at the regulon level with the deletion and overexpression mutants of *oxyR* (*oxyRΔ3* and *oxyR2* respectively). Results are shown in Fig 3C. As expected, the strain overexpressing *oxyR (oxyR2)* was protected better than the wildtype. The deletion mutant *oxyRΔ3* was found significantly susceptible (p = 0.002), like those of *ΔkatE* and *ΔahpC* mutants. This indicates the overall involvement of the *oxyR* system in defense against oxidative stress produced by plasma-charged aerosols.

### Effect of catalase and other scavengers

**Fig 4:**
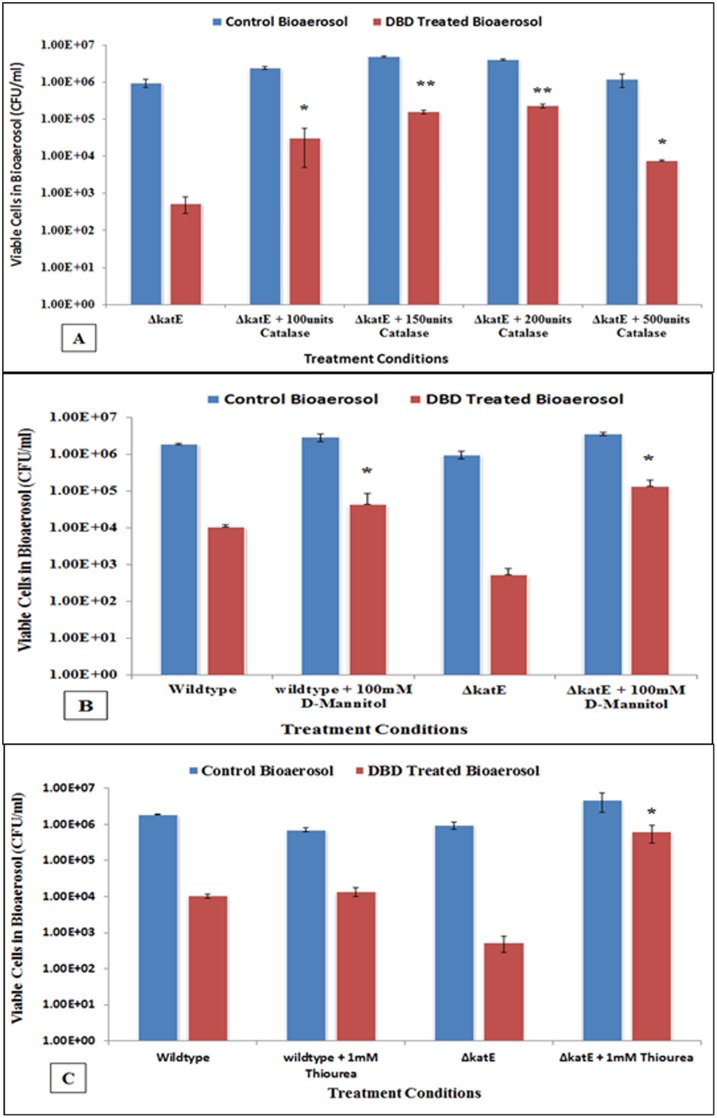
The colony assays showing effect of scavengers on rescue of plasma charged aerosol post-treated *E*. *coli* wildtype and mutants. **(A)** Catalase, the specific scavenger of H_2_O_2_ was able to significantly protect mutant (*ΔkatE*) from inactivation. **(B)** A non-enzymatic ROS scavenger, D-mannitol was significantly able to rescue wildtype and *ΔkatE* cells from inactivation as compared to their corresponding non-scavenged conditions. **(C)** Thiourea, a wildly used ^.^OH scavenger was able provide significant protection to deletion mutant (*ΔkatE*) but not the wildtype *E*. *coli*, suggesting that intracellular ^.^OH pool created in hydroperoxidase deficient cells during post-exposure to plasma charged aerosols are effectively scavenged, and possibly inhibit further Fenton chemistry generating hydroxyl radicals. This also demonstrates that *katE* is an important mediator of survival in bioaerosolized *E*. *coli* under such condition. (* indicates *p* <0.05 against their corresponding conditions without scavenger. ** indicates *p* <0.05 against remaining concentrations of catalase).

Testing of the gene knockout mutant strains has indicated the involvement of peroxidative stress produced by plasma. The addition of exogenous catalase has been shown to protect bacteria from exogenous peroxidative stress [44]. Of the catalase gene knockouts, *ΔkatE* was chosen to analyze the effect of external catalase, since it had shown significantly higher susceptibility than the wildtype. Various concentrations of the catalase were tested as pre- and post- treatment protective mechanism. Addition of the catalase prior to nebulization and then DBD treatment produced no significant change in the inactivation. Post-treatment addition consisted of collecting the treated aerosol in a solution containing catalase. The results indicate that for all of the concentrations of catalase tested, a significant protection compared to wildtype treatment was observed (Fig 4A). For increasing amount of catalase, the protective effect increased, but complete protection was not achieved. A plateau observed at 200 units of catalase, providing significant protection (p = 0.0001), and then thereafter (500 units) did not follow trend, but was still significant (p = 0.001). This validates our earlier statement about the involvement of peroxide produced from plasma-charged aerosols in bacterial inactivation. A complete protection was not observed and this can be indicative of the involvement of other species produced by plasma. Two non-enzymatic scavengers of ROS, D-mannitol and thiourea were also used. D-mannitol has been shown to defend against peroxidative stress [39], and thiourea also reportedly has been used as an ^.^OH scavenger [40]. The ^.^OH is one of the major ROS that is deleterious to bacteria. The two scavengers are not endogenous to bacterial cell. Therefore, the wildtype was also tested to determine their efficiency in protection. Both scavengers were added to the test strains pre- and post- treatment of plasma. Pre-treatment in these experiments provided no significant protection, but for post-treatment, the addition of mannitol protected the catalase deficient strain like that of wildtype strain (Fig 4B). Of the various concentrations of the scavenger added, the greatest protection was observed for 100mM (p = 0.001). The results with thiourea also exhibited the similar trend to mannitol mediated protection (Fig 4C). One millimolar (mM) of thiourea (pre-optimized) provided a significant protection to *ΔkatE* strain (p = 0.0028). The protective effect to wildtype was not significant. The protective effect decreased for higher concentration of thiourea. For 100mM thiourea addition, there was 3 logs reduction in untreated as well as treated bioaerosol (data not shown). This effect might be attributed to the fact that thiourea becomes toxic to cells at high concentrations [40].

### Activation of heat shock proteins in bacteria during plasma treatment of bioaerosols

**Fig 5:**
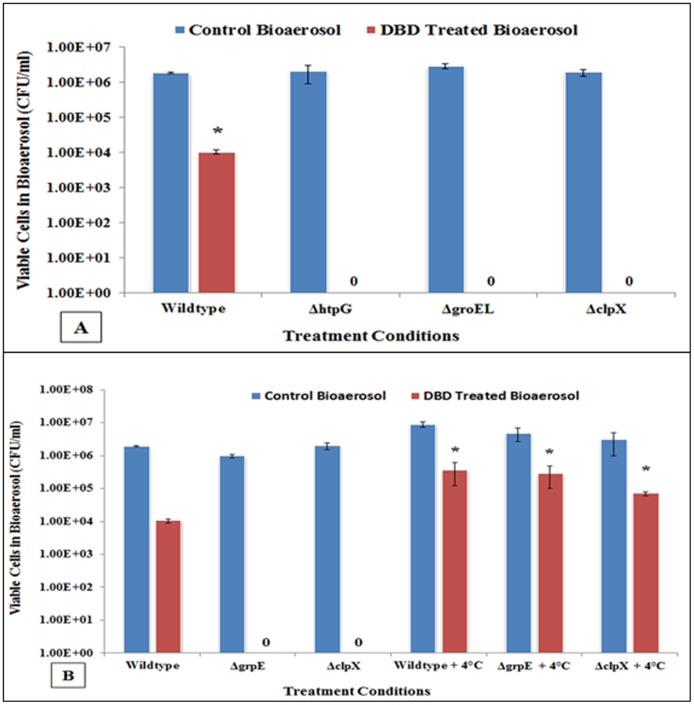
The colony assays demonstrating the effect of plasma charged aerosols on heat shock protein (hsp) deficient *E*. *coli* derivatives. **(A)** All *hsp* deficient *E*. *coli* (*ΔhtpG*, *ΔgroEL*, *ΔclpX*) showed complete inactivation of cells at ambient temperature. (*, p <0.05 against corresponding untreated bioaerosols) **(B)** When the treatments with plasma charged aerosols were carried out at cold temperature (4°C), all *hsp* deletion mutants and wildtype *E*. *coli* cells significantly survived. (*, p <0.05 against corresponding ‘no scavenging’ conditions). Together, these findings demonstrate that *hsp* activation occurs in *E*. *coli* cells in response to DBD charged aerosol exposure at room temperature, *htpG*, *groEL*, *clpX* and *grpE* are directly involved cell survival, and cold temperature scavenge intracellular localized heating effect and protect these specific mutants.

Environment induced stress in *E*. *coli* especially severe oxidative and/or heat shock, induces heat-shock proteins (hsp). When the cells start experiencing stress or rise in temperature to a point where protein’s three-dimensional structure and spatial orientation starts compromising and proteins start to unfolding, the heat-shock proteins are induced and interact with those proteins to hold them together to keep the functional integrity of cell. Earlier we observed that non-thermal plasma treatment of bioaerosol generate oxidative stress in bacterial cell, and therefore we decided to investigate the survival response of *hsp* deletion mutants and wildtype *E*. *coli*. As the representative of heat-shock protein management pathway, we selected deletion mutants, *ΔhtpG*, *ΔgroEL ΔclpX* and *ΔgrpE* (S1 Table). DBD treatment of wildtype and all *hsp* deletion mutants of *E*. *coli* were significantly inactivated as compared to their no-treatment controls (P = < 0.05) (Fig 5A). In fact, all bioaerosolized deletion mutants were completely inactivated during exposure DBD and about 2 logs reduction in CFU of wildtype *E*. *coli* was observed. This indicated that heat-shock proteins are involved in offering resistance toward inactivation of *E*. *coli*, and it is possible that there might be a sufficient heath stress generated inside cells, along with oxidative stress, or they might be related in protecting cells against plasma exposure. Therefore we decided to scavenge heating effect by conducting whole experiment at 4°C. It was surprising to observe that all the strains of *hsp* deletion mutant and wildtype were significantly protected as compared to their corresponding conditions carried out at ambient/ room temperature (Fig 5B). The wildtype strain had also shown a significant protection (p = <0.05) at cold temperature. These experiments further confirm that heat shock proteins are involved in stress management, and are induced during plasma treatment of bioaerosolized form of *E*. *coli*.

## Discussion

Recently, plasma technology is explored as a new approach to inactivation of bioaerosols. Having trial demonstration of the efficacy of a large device DBD system for bioaerosols inactivation, a miniaturized, small system was designed and tested in our laboratory (Fig 1) to learn about the mechanism of inactivation of aerosolized bacteria. The preliminary results indicate that the proposed device-generated point to point DBD plasma was highly efficient in inactivation of both the smaller load (4 logs) and larger load (7 logs) of aerosolized bacteria (Fig 2) and inactivation was plasma energy-dependent. The samples cultured on TSA plates were incubated for extended period (up to 96 hours) to find out whether bacteria undergo inactivation or dormancy, and findings confirmed that bacteria were completely inactivated. The lethal and sublethal doses were determined to find out whether the total bacterial cell population is inactivated (S1 Fig; Fig 2D), and the sublethal dose would be sufficient to activate stress induced key regulators. Findings supports that lethal dose treatment does not allow bacterial cells to survive (findings similar to negative control; aerosols alone) whereas sublethal dose treated cells re-grew similar to untreated bioaerosol, and showed similar pattern of cellular respiration during XTT assay (S2B Fig). A significant membrane depolarization of *E*. *coli* cells (S2A Fig) by both Low and high plasma energy doses was obvious. Membrane potential detected in most bacteria by the BacLight^™^ Bacterial Membrane Potential kit, and the magnitude varies with species, and response may not appear to be proportional to depolarizing agent (Molecular Probe, Inc.)

The system was tunable and the efficacy found dependent upon the amount of bacteria load, generation rate of bioaerosols, and flow rate of bioaerosol. The organisms selected for aerosolization were clinically relevant to hospital environment, including multidrug resistant MRSA (Fig 2B). Inactivation of environmental bacterial species (such as *Pseudomonas fluorescence* and *Bacillus subtilis*) are demonstrated using different device model generating nonthermal plasma [13], but not the clinically relevant species or a point to point DBD device used in these studies. Plasma-charged aerosols are fast acting and offer an excellent alternative for efficient air decontamination technology [13]. Antibiotic resistant pathogens such as MRSA USA 300 and MRSA USA400 are community-acquired MRSA (CA-MRSA) but their presence started noticing in hospital premises and replacing hospital-acquired MRSA (HA-MRSA) [45]. The inactivation treatment of bioaerosols generated in hospitals or healthcare centers therefore would be effective strategy to contain and prevent spreading dangerous pathogen outside environment. This technology has direct application in hospital environment and clinical setting where sterilization of air carrying aerosolized bacteria is required.

There are two key regulatory oxidative stress management pathways, and these are mediated by superoxide dismutase (sod) and catalase (cat). The superoxide dismutase mutants did not show significant change toward plasma-charged aerosols as compared to wildtype. This may be partly due to the fact that O_2_^-^ permeates the bacterial membrane poorly as compared to H_2_O_2_ [46], and/or possibly the superoxide anion might be getting converted to the more stable species H_2_O_2_ within the water in the bioaerosol droplet. The regulon oxyR play a major role in balancing cellular redox states condition, which is predominantly sensitive to hydrogen peroxide than superoxide, and induce scavengers such as H_2_O_2_ scavengers and other defensive enzymes [34]. OxyR protein is directly sensitive to oxidation, and only oxidized OxyR is capable of transcription. Even the small amount [0.025 μM] of H_2_O_2_ is sufficient to oxyR mediated oxidative damage in *E*. *coli* [47]. Our findings indicate that plasma-charged aerosols produce predominantly peroxidative stress in *E*. *coli*, and the responses indicate that *katE* deletion mutant was significantly susceptible toward inactivation. A mutant of *E*. *coli* that lacks catalase or peroxidase does not degrade hydrogen peroxide at significant rate, but however, surprisingly the mutant defective in both catalases (*ΔkatE*, *ΔkatG*) typically exhibit no adverse effect on cell survival. Under these circumstances, another scavenger, alkyl hydroperoxidase reductase (Ahp) assume major detoxification role in double mutant (*ΔkatEΔkatG*) [48]. Ahp enzyme appears to be almost exclusively involved in endogenous low concentrations of H_2_O_2_ in *E*. *coli* [48–50], and is under control of *oxyR*. In present studies, *ΔahpC* deletion mutant was significantly more susceptible to plasma-charged aerosols, suggesting the involvement of Ahp scavenger. Interestingly, *katG* mutant did not show any significant change in inactivation from wildtype; and this may be because both *oxyR* and *rpoS* pathways are functional even in absence of *katG*. The loss of either *katE* or *ahpC* seems to have overwhelmed the defenses of bacteria, leading to greater susceptibility to cell death.

*KatE* activation is central to many ROS managing pathways, including the stress induced by changes in pH, temperature and heat shock, osmotic pressure or photosensitization [51]. The hydroperoxidase (product of *katE*) is also influenced by other intracellular metabolic products, ROS and redox state, and the stage of cellular growth phase [51]. In our studies, all three ROS scavenger viz. catalase, D-mannitol, and thiurea were able to protect *E*. *coli katE* deletion mutant from plasma-charged aerosol significantly as compared to corresponding conditions without scavenger, and suggests that *katE* play a critical role in preventing cells from inactivation. While catalase is a specific scavenger of H_2_O_2_, D-mannitol and thiourea are wildly used as scavenger of free hydroxyl radicals as well [52–54]. We hypothesize that hydroxyl radicals generated in the plasma are transported into the aerosol aqueous phase where they may then recombine, thus increasing hydrogen peroxide concentration, a more stable and predominant species in proposed plasma-charged aerosol, or the hydroxyl scavenger suppress hydroperoxide formation in aerosol. Former is more likely, and indicative of the activation of *katE*.

We hypothesized that during DBD exposure of aerosols a transient localized heat stress might be generating in bioaerosolized *E*. *coli* cells, and hence decided to look at the activation of heat-shock proteins (hsp). The activation of both *ahpC* and *katE* are known to induce heat shock proteins [55,56]. Heat stress is usually sensed through the misfolding and/or unfolding of outer membrane porin precursor proteins. Eventually, the induced hsp act as the major chaperons which try to protect critical cellular proteins from unfolding by directly interacting with these proteins to hold together and keep them functional. The induction of type and amount of hsp also depends upon the cellular response and type of stress, and provide a shield by post-translational modification of proteins. Heat shock protein GroEL (also known as HSP60) is one of the major chaperons found in cytoplasm of *E*. *coli*, sensitive to elevated temperature stress and hydrogen peroxide mediated oxidative stress, and principally known to regulate the transcription of the major heat shock proteins and the molecular chaperons required for heat-shock response [51]. In our studies, deletion mutant, *ΔgroEL* found extremely susceptible to plasma-charged aerosols, and a complete inactivation of 7 logs CFUs was observed, which indicates a direct involvement of *groEL* in cellular protection. Another heat shock protein from HSP100 chaperon family, ClpX is involved in ATP-driven regulated proteolysis, which adjusts cellular protein pool by removal of non-functional damaged proteins and play a vital role in restoring cell homeostasis [57]. Protein folding is known to be mediated principally by the ribosome-associated trigger factor, the chaperonin system GroEL/GroES, and the Hsp70 system (DnaK/DnaJ/GrpE), of which the latter is most adaptable system in *E*. *coli*. GrpE is nucleotide exchange factor which implicate in regulation of sigma factor σ^32^ activity and σ^32^ degradation. Inactivation or mutation of *grpE* induces heat shock response through increase in σ^32^ stability and activity (a positive regulator of hsps transcription). Thus, GrpE protein behave as a negative modulator that mediate degradation as well as repression of σ^32^ stability, and hence, GrpE-dependent repair during cell survival [58]. Another highly conserved, abundant chaperon is HtpG (Hsp70 homolog), encoded by *htpG* in *E*. *coli*, which facilitate protein folding and remodeling by activating ATPase activity in stressed cells via associating with ribosomal protein L2, and is essential in thermal stress management [59]. In our studies, the deletion mutants, *ΔgrpE*, *ΔhtpG*, *ΔgroEL*, and *ΔclpX* were all extremely susceptible to plasma-charged aerosols, and resulted in complete inactivation of 7 logs CFUs. The heat shock stress was scavenged and the tested *E*. *coli* deletion mutants and wildtype strain found significantly protected when the bioaerosols treatment with DBD plasma was carried out at 4°C. Thus it is evident that the transcription of proposed candidate *hsp* genes are involved in *E*. *coli* cell protection when exposed to DBD plasma-activated aerosol generated stress, and the survival is dependent on critical Hsp proteins such as GrpE, HtpG, GroEL, ClpX, and the antioxidant enzymes KatE and AhpC. Further studies are required to correlate in detail the complex system of oxidative stress, heat shock stress and chaperonin system in plasma-charge treated aerosolized *E*. *coli*.

## Supporting information

S1 Fig *E*. *coli* responses in bioaerosol form to no treatment or treatment with sublethal dose and lethal dose of DBD. (TIF)

S2 Fig Changes associated with membrane potential and cellular respiration in *E*. *coli*. (TIF)

S1 Supporting Information Characteristics of waveform, voltage, current and pulse. (PDF)

S1 Table List of *Escherichia coli* strains used in present studies. (PDF)

## Abbreviations

Cat: catalase
CFU: colony forming unit
DBD: dielectric-barrier discharge
plasma: nonthermal DBD plasma
SOD: superoxide dismutase
XTT: 2,3-bis-(2-methoxy-4-nitro-5-sulfophenyl)-2H-tetrazolium-5-carboxanilide
