# Supplementary figures and images for: Involvement of multiple stressors induced by non-thermal plasma-charged aerosols during inactivation of airborne bacteria
Source: PLoS One. 2017 Feb 6;12(2):e0171434. doi: 10.1371/journal.pone.0171434 (PMC5293192; doi:10.1371/journal.pone.0171434)

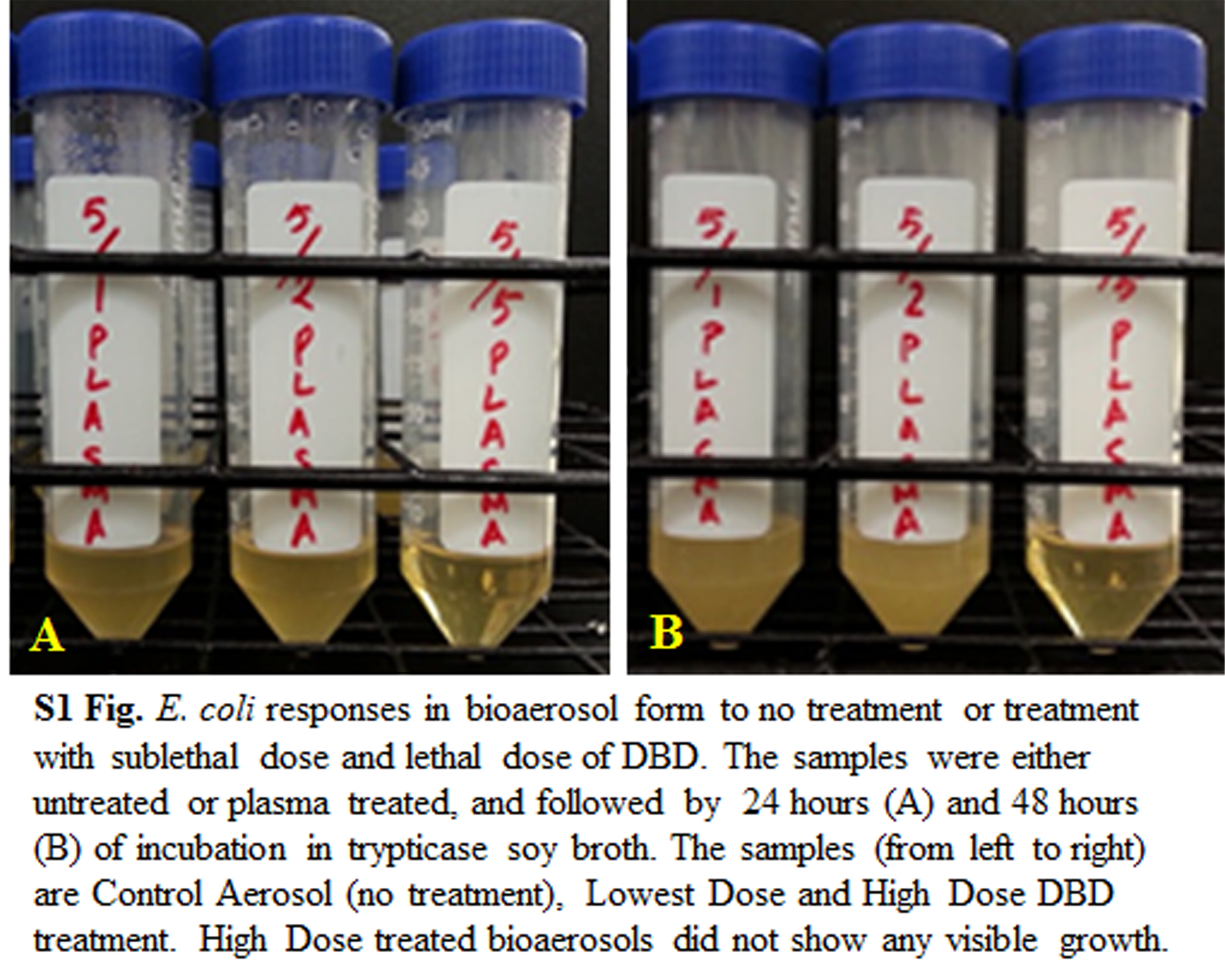

Supplement: S1 Fig — (TIF) [file pone.0171434.s001.tif]

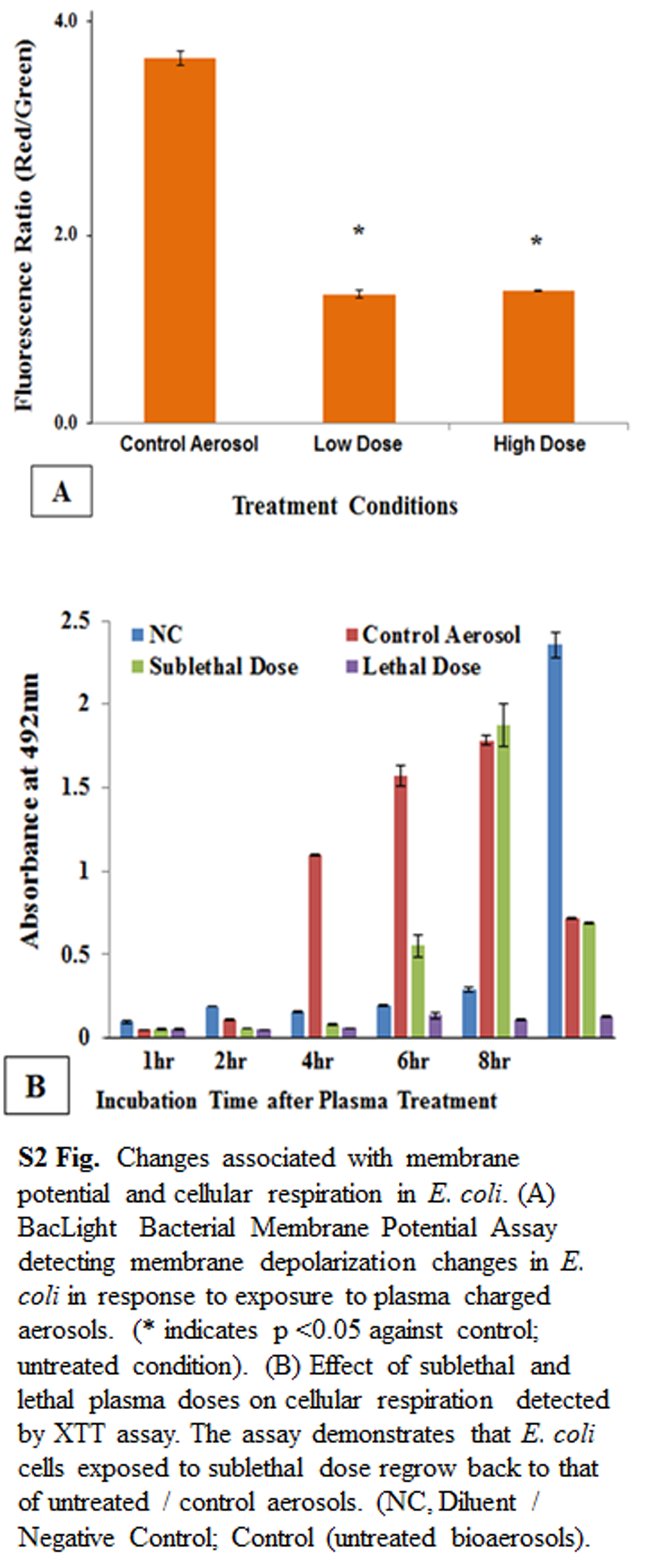

Supplement: S2 Fig — (TIF) [file pone.0171434.s002.tif]
