# Supplementary material for: Involvement of multiple stressors induced by non-thermal plasma-charged aerosols during inactivation of airborne bacteria
Source: PLoS One. 2017 Feb 6;12(2):e0171434. doi: 10.1371/journal.pone.0171434 (PMC5293192; doi:10.1371/journal.pone.0171434)
Supplement: S1 Supporting Information — (PDF) [file pone.0171434.s003.pdf]

S1 Supporting Information. Characteristics of Waveform, Voltage, Current and Pulse.

Waveform.

Output waveform measurements were made and current and voltage monitored for High Voltage Microsecond Pulse Generator used in the present studies. The model parameters of output pulse waveform, and measured and modeled waveforms are shown below (this waveform is referred to as a single "pulse").

Table (a). The model parameters of output pulse waveform.

MODEL PARAMETERS		
t_{OFFSET} =	10.000E-6	s
V_0 =	14,000.00	V
f_1 =	36.000E+3	Hz
f_2 =	250.000E+3	Hz
$\beta = A_2 / A_1$ =	0.15	
α =	1.40E+04	s^{-1}

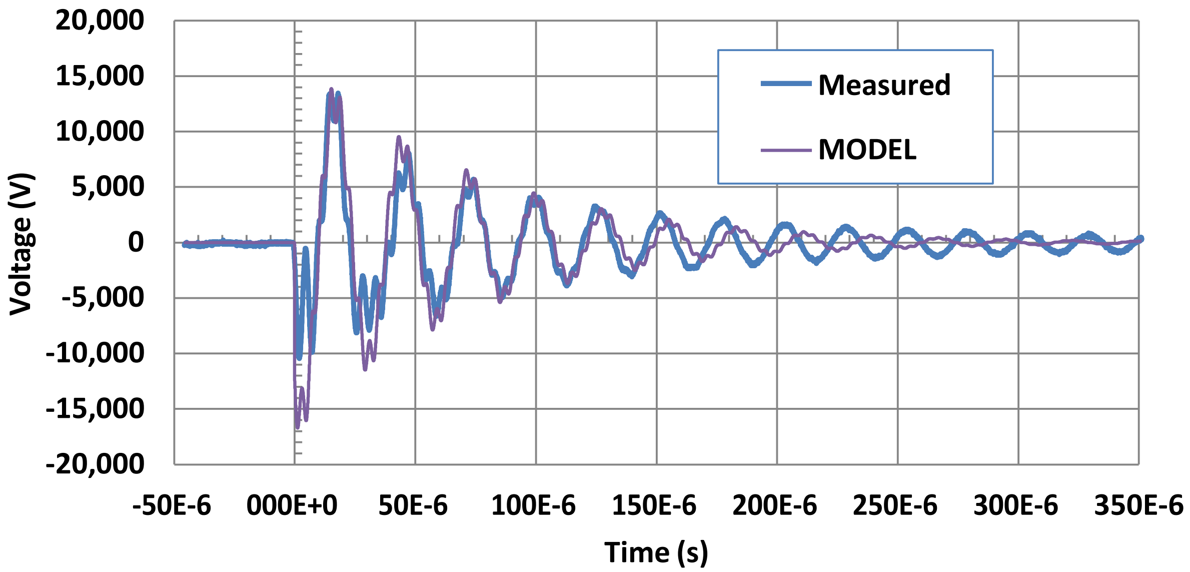

Figure (i). The measured and modeled waveforms (single "Pulse").

Features of Pulse Train.

The frequency control (“FREQ”; DIAL 0-9) sets the number of pulses produced in a 10 ms period. The number of pulses in this pulse train varies nonlinearly with the DIAL setting and the length of the pulse train varies from 4 ms to almost 10 ms. The “FREQ” control timing calibration is shown in Table (b) and Figure (ii).

Table (b): The frequency control (“FREQ” dial) settings and timing.

FREQ DIAL	# Pulse in 10 ms window (Pulse Train)		Pulse Spacing (ms)	Total Pulse Train Time (ms)	Effective Frequency (Hz)
	Min	Max			
0	0	1	n/a	n/a	100
1	1	2	4.0000	4.0000	250
2	2	3	4.0000	4.0000	250
3	5	5	2.0000	8.0000	500
4	10	11	0.9690	8.7210	1032
5	15	16	0.6486	9.0804	1542
6	22	22	0.4636	9.7356	2157
7	27	27	0.3706	9.6356	2698
8	30	31	0.3238	9.3902	3088
9	36	36	0.2778	9.7230	3600

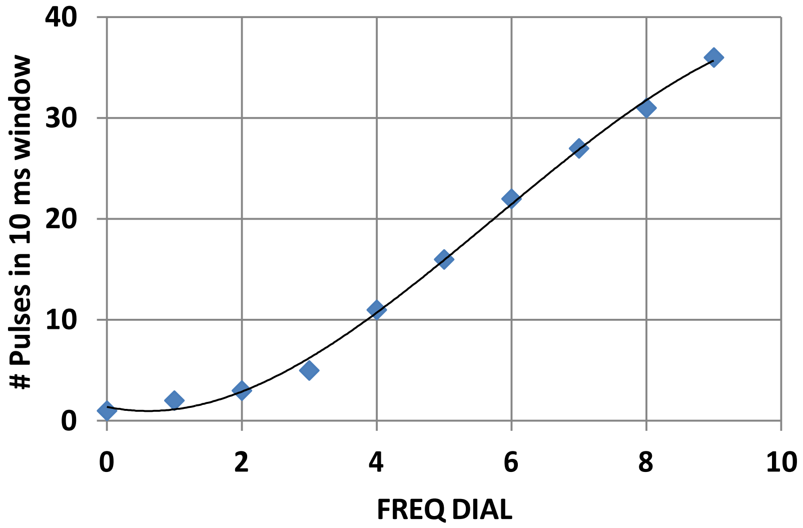

Fig (ii): Number of pulses in a pulse train vs. frequency dial (FREQ DIAL) setting.

<u>Amplitude</u>.

The magnitude of the output voltage into the plasma system (open load) is set and shown by the "V_{P-P}" in Table (c) and Figure (iii). The peak-to-peak voltage increases nonlinearly from 17.5 to 25.9 kV over the full range of settings, but increases only 3.2 kV (20.5 to 23.7 kV) in the 2 to 9 dial settings range.

Table (c). The "V_{P-P}" control of output voltage magnitude.

V_{P-P} DIAL (0-9)	V_{P-P} (kV)	V_P (kV)	I_{P-P} (mA)	I_P (mA)
1	17.5	8.5	474	247
2	20.5	10.1	458	231
3	20.9	10.5	442	215
4	20.7	10.3	438	211
5	21.1	10.7	450	223
6	23.5	13.1	575	311
7	24.3	13.9	603	323
8	24.5	14.1	474	247
9	23.7	13.5	442	211
0	25.9	15.5	515	279

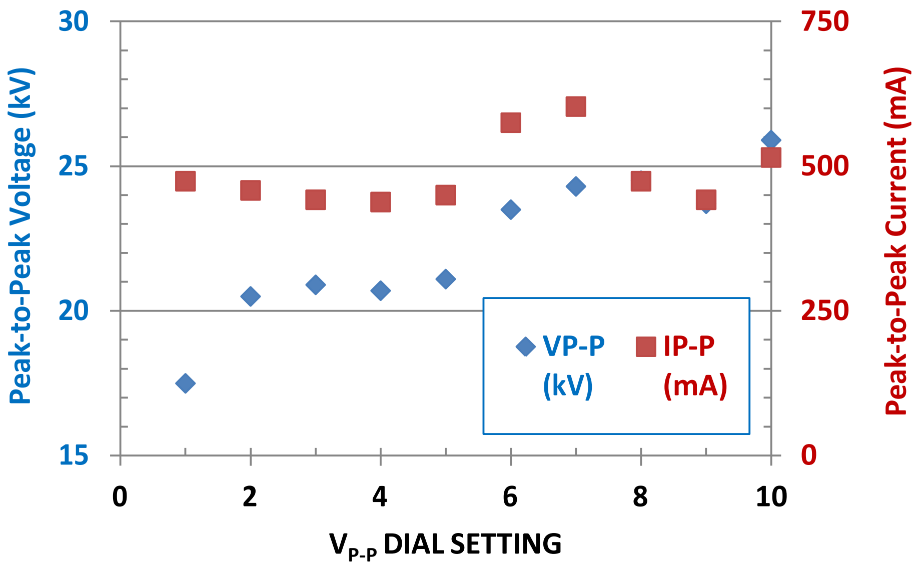

Figure (iii). Peak-to-peak voltage and current output vs. V_{P-P} DIAL setting. (DIAL setting "0" corresponds to "10" on graph.)
