# Supplementary material for: Involvement of multiple stressors induced by non-thermal plasma-charged aerosols during inactivation of airborne bacteria
Source: PLoS One. 2017 Feb 6;12(2):e0171434. doi: 10.1371/journal.pone.0171434 (PMC5293192; doi:10.1371/journal.pone.0171434)
Supplement: S1 Table — (PDF) [file pone.0171434.s004.pdf]

S1 Table. List of *Escherichia coli* strains used in present studies.

Strain No.	Relevant genotype	Pathway deletion mutant	Reference
BW25113	Wild type	-	(1)
3144	*BW25113 ΔsodA*	Superoxide dismutase	(2)
3145	*BW25113 ΔsodB*	Superoxide dismutase	(2)
3156	*BW25113 ΔsodAΔsodB*	Superoxide dismutase	(2)
3157	*BW25113 ΔkatG*	Catalase/Peroxidase	(2)
3202	*BW25113 ΔkatE*	Catalase/Peroxidase	(2)
3201	*BW25113 ΔkatGΔkatE*	Catalase/Peroxidase	(2)
3200	*BW25113 ΔahpC*	Catalase/Peroxidase	(2)
TA4110	*oxyR2, oxyR Constitutive*	Oxy Regulon	(3)
TA4112	*oxyRΔ3, oxyR Deletion*	Oxy Regulon	(3)
JW4103-1	*groEL: groL768(del)::kan*	groES(hsp10)/groEL(hsp60)	*
DA16	*grpE: grpE280*	dnaJ(hsp40)/dnaK(hsp70)/grpE(hsp60)	*
JW0462-1	*HtpG: htpG757(del)::kan*	htpG(hsp90)	*
JW0428-1	*clpX: clpX724(del)::kan*	clpABX(hsp100)	*

(*, Strains obtained from *E. coli* Genetic Stock Center, Yale University, New Haven, CT)

References:

(1) Baba, T.; Ara, T.; Hasegawa, M.; Takai, Y.; Okumura, Y.; Baba, M.; Datsenko, K. A.; Tomita, M.; Wanner, B. L.; Mori, H., Construction of *Escherichia coli* K-12 in-frame, single-gene knockout mutants: the Keio collection. *Molecular systems biology* **2006,** *2*, 2006-08.

(2) Wang, X.; Zhao, X., Contribution of oxidative damage to antimicrobial lethality. *Antimicrobial agents and chemotherapy* **2009,** *53*, (4), 1395-402.

(3) Christman, M. F.; Storz, G.; Ames, B. N., OxyR, a positive regulator of hydrogen peroxide-inducible genes in *Escherichia coli* and *Salmonella typhimurium*, is homologous to a family of bacterial regulatory proteins. *Proceedings of the National Academy of Sciences of the United States of America* **1989,** *86*, (10), 3484-8.
